# G6PD deficiency in malaria endemic areas of Nepal

**DOI:** 10.1186/s12936-020-03359-6

**Published:** 2020-08-12

**Authors:** Baburam Marasini, Bibek Kumar Lal, Suman Thapa, Kiran Raj Awasthi, Bijay Bajracharya, Pratik Khanal, Sanjeev Neupane, Shambhu Nath Jha, Sanjaya Acharya, Smriti Iama, Madan Koirala, Dinesh Koirala, Suresh Bhandari, Ram Kumar Mahato, Arun Chaudhary, Pramin Ghimire, Rahachan Gharti Magar, Rajan Kumar Bhattarai, Gornpan Gornsawun, Pimsupah Penpitchaporn, Germana Bancone, Bhim Prasad Acharya

**Affiliations:** 1 Epidemiology and Disease Control Division, Department of Health Services Government of Nepal, Teku, Kathmandu, Nepal; 2 Save The Children, Global Fund, Airport, Shambhu Marg, Kathmandu, Nepal; 3 Epidemiology and Disease Control Division/Program Management Unit (Global Fund/SCI), Teku, Kathmandu, Nepal; 4 Institute of Medicine, Tribhuvan University, Kathmandu, Nepal; 5 Shoklo Malaria Research Unit, Mahidol-Oxford Tropical Medicine Research Unit, Faculty of Tropical Medicine, Mahidol University, Mae Sot, Thailand; 6 Centre for Tropical Medicine and Global Health, Nuffield Department of Medicine, University of Oxford, Oxford, UK

**Keywords:** G6PD deficiency, Malaria, Nepal, *Plasmodium vivax*, Primaquine, 8-Aminoquinolines

## Abstract

**Background:**

Glucose-6-phosphate dehydrogenase (G6PD) deficiency is currently a threat to malaria elimination due to risk of primaquine-induced haemolysis in G6PD deficient individuals. The World Health Organization (WHO) recommends G6PD screening before providing primaquine as a radical treatment against vivax malaria. However, evidence regarding the prevalence and causing mutations of G6PD deficiency in Nepal is scarce.

**Methods:**

A cross-sectional, population-based, prevalence study was carried out from May to October 2016 in 12 malaria-endemic districts of Nepal. The screening survey included 4067 participants whose G6PD status was determined by G6PD Care Start™ rapid diagnostic test and genotyping.

**Results:**

The prevalence of G6PD deficiency at the national level was 3.5% (4.1% among males and 2.1% among females). When analysed according to ethnic groups, G6PD deficiency was highest among the Janajati (6.2% overall, 17.6% in Mahatto, 7.7% in Chaudhary and 7.5% in Tharu) and low among Brahman and Chhetri (1.3%). District-wise, prevalence was highest in Banke (7.6%) and Chitwan (6.6%). Coimbra mutation (592 C>T) was found among 75.5% of the G6PD-deficient samples analysed and Mahidol (487 G>A) and Mediterranean (563 C>T) mutations were found in equal proportions in the remaining 24.5%. There was no specific geographic or ethnic distribution for the three mutations.

**Conclusions:**

This study has identified populations with moderate to high prevalence of G6PD deficiency which provides strong evidence supporting the WHO recommendations to screen G6PD deficiency at health facility level before the use of primaquine-based radical curative regimen for *Plasmodium vivax*.

## Background

Malaria remains a global problem with an estimated 228 million cases occurring globally and 405,000 deaths in 2018. It is still endemic in 91 countries with about half of the world’s population at risk, particularly those living in lower-income countries [1]. Ending the epidemic of malaria, AIDS, tuberculosis and other neglected tropical diseases by 2030 is related to target 3.3 of the Sustainable Development Goals. The Asia–Pacific countries, including Nepal, have shown their commitment to regional elimination of malaria by 2030 [2]. Although the global incidence of malaria has decreased by 41% between 2000 and 2015, fewer than half of the countries are on track to achieve the global strategy target of 40% case and mortality reduction by 2020 [1, 3, 4].

The presence of G6PD deficiency among the population is a huge challenge for malaria treatment due to the associated haemolytic risk with radical treatment using 8-aminoquinolines. G6PD deficiency is the most common human enzyme defect and is mostly asymptomatic. However, G6PD-deficient individuals may suffer from acute haemolytic anaemia (AHA) when exposed to several oxidative triggers, such as fava beans, infections or anti-malarial drugs such as primaquine. AHA is the most common manifestation of this deficiency associated with intra and extra-vascular haemolysis, jaundice and haemoglobinuria with acute renal failure as the most severe outcome [5–11]. Factors affecting individual haemolytic risk include drug dose and drug metabolization (dependent from CYP2D6 in primaquine), G6PD phenotype and genotype, and haematologic picture, particularly red blood cell age distribution [2, 10, 12]. At population level, males are historically considered more susceptible to haemolysis because the deficient phenotype is more prevalent among them, but recent evidence shows that heterozygous women with intermediate G6PD phenotypes are also at risk of severe haemolysis [13, 14].

About 400 million people are estimated to be G6PD deficient worldwide [5, 15–18], with a geographical distribution similar to that of malaria [5, 6, 19] but also with high variability within countries and regions. The affected population in malaria-endemic countries is estimated to be 220 million males and 133 million females [12, 16]. Sub-Saharan Africa has the highest average estimated prevalence (7.5%) followed by Middle East (6.0%) and Asia (4.7%). The prevalence is lowest for Pacific (2.9%), Americas (3.4%) and Europe (3.9%) [17].

Studies on G6PD deficiency among a small number of individuals of Nepalese origin were conducted as early as 1960s in Calcutta [20]. Studies conducted until the early 2000s, among Nepalese healthy individuals, found extreme variability in G6PD prevalence among ethnic groups and geographic regions; Tharus living in Banke and Bardiya district showed an exceptionally high prevalence (14.0%) compared to most of other ethnicities and regions. The mutation responsible for the deficiency was identified in one study to be the Mediterranean variant [21–24]. In a recent and first population-based study conducted in six high-risk malaria-endemic districts in Nepal in 2013 [25], G6PD deficiency screening was carried out among 1341 individuals using two screening tests: BinaxNOW and CareStart™. The prevalence of G6PD deficiency was found to be 7.2% according to BinaxNOW and 6.0% by CareStart™. A higher prevalence was observed among males of Rajbanshi and Tharu ethnic groups, and in Jhapa and Morang districts.

Nepal has a long history of malaria control programmes starting from 1954 [26] and its commitment to malaria elimination has been reflected in Nepal Malaria Strategic Plan (2014–2025) which aims for malaria-free Nepal by 2026 [26, 27]. The high risk areas of malaria in the country are the foothills with river belts, forest fringe areas in the Tarai, hill river valleys and inner Tarai areas [26]; for recent maps of malaria endemicity see [28]. In Nepal, malaria is decreasing sharply (from 12,750 cases in 2002 to 991 in 2016) and it is caused predominantly by *Plasmodium vivax* [26, 29]. However, *P. vivax* cases have been recorded in non-endemic areas representing a challenge for malaria elimination efforts. The Epidemiology and Disease Control Division (EDCD) has adopted the World Health Organization (WHO) policy to screen malaria patients for G6PD deficiency in its National Malaria treatment protocol published in 2015 [30].

Understanding the burden of G6PD deficiency and identifying vulnerable populations is crucial for the success of the malaria control programme in Nepal. In this regard, this study aims to estimate the prevalence of G6PD deficiency and to describe the underlying mutations in areas where malaria transmission is endemic. It is anticipated that the study findings will inform strategic plans for the malaria elimination programme in Nepal.

## Methods

A cross-sectional prevalence survey was conducted among 4067 study participants of 12 malaria-endemic districts of Nepal. Data were collected from May 2016 to October 2016. The study population comprised both male and female participants above 6 years of age and residing in the study districts.

### Study sites

The study districts were Banke, Bardiya, Chitwan, Dadeldhura, Kailali, Kanchanpur, Kapilvastu, Makwanpur, Nawalparasi, Rautahat, Sindhuli, and Surkhet. These districts were selected according to malaria endemicity data based on the malaria micro-stratification report 2013 (http://edcd.gov.np/resource-detail/report-on-malaria-microstratification-2013).

### Sampling method

Two-stage, cluster-sampling method was used for drawing participants in the sample. Among the 1254 Village Development Committees (VDCs), 54 were identified to be at high risk of malaria based on the malaria micro-stratification report 2013. These VDCs were further divided into clusters and 30 of them were selected using systematic random sampling with probability inclusion proportional to size (PPS) method from the VDC.

The field teams visited each selected cluster to prepare a list of households for the study and 132 households were selected by systematic random sampling method from each cluster. From this list, a male participant was selected from the first and second household and a female participant was selected from the third household; this was repeated for every block of three successive households resulting in the selection of a total of (30 × 132) = 3960 participants with a male to female ratio of 2:1. The total final sample size of the study was 4067 due to additional samples taken in some sites.

### Data and samples collection methods

A structured questionnaire was used for data collection, including socio-demographic characteristics. Interviews were conducted in Nepali language by researchers of the same gender of interviewed participants. Screening of G6PD status was done on capillary blood using CareStart™ (Access Bio. Inc., New Jersey, USA). CareStart™ retraining was supported by investigators of the Shoklo Malaria Research Unit (SMRU) prior to survey activities. The test was run following manufacturer’s instructions. Test interpretation was based on colour change in the reading window, with a pink purple colour interpreted as ‘G6PD normal’ result and a faint or no colour development interpreted as ‘G6PD deficient’ result. Blood for human genotyping was spotted on filter papers, dried, and stored at − 20 °C.

### Study variables

The study variables were socio-demographic variables (age, gender, ethnicity, permanent residence), previous history of malaria and G6PD screening results. Since the ethnicity and caste classification of Nepal is extremely complex [31], both the ethnicity and the Ministry of Health larger group classification were used in the analysis.

Data were collected in the field by district level laboratory staff, peripheral level laboratory and health workers supervised by District Public Health Office (DPHO) team and EDCD team. Data were quality controlled to assure uniformity across all sites.

### Laboratory analysis

Dried blood spots of subjects who tested deficient were shipped on ice packs to SMRU in Mae Sot (Thailand). DNA was extracted by mini-column (Favorgen Biotech, Taiwan) or saponin-chelex method for PCR–RFLP and gene sequencing from exon 2 to exon 13 of G6PD gene. Primer list and PCR conditions are reported in Additional file 1: Table S1 and S2. Amplified fragments were sent to external sequencing platforms and results were analysed by CLC Main Workbench 6 (Qiagen Bioinformatics). Based on published literature at the time of the study, Mediterranean (563 C>T) and Coimbra (592 C>T) mutations were expected to be found in the population, so all samples were analysed first for exon 6 which contains both variants and Mahidol (487 G>A). When samples were found to harbour no variants in exon 6, the other exons were analysed.

### Data management and statistical analysis

Survey data were entered in Microsoft Excel and then transported into IBM SPSS version 20. Double entry was done to ensure accuracy. Prevalence of G6PD deficiency was calculated from the pooled number of subjects with deficient or suspected deficient phenotype over the total of subjects included. Allelic frequencies were calculated as the number of mutated alleles (one in hemizygous males, one in heterozygous females and two in homozygous mutated females) over the total number of alleles (where females contribute two alleles each and males one allele each). Allelic frequencies among groups were compared by Chi square test. A P < 0.05 was considered significant.

## Results

The screening of G6PD deficiency was done among 4067 participants. Ten participants whose data were missing were removed from further analysis, thus 4057 participants were included in the final analysis.

### Demographic characteristics of the participants

The demographic characteristics of the participants are summarized in Table 1. As per study design, almost two-thirds of the participants (2564, 63.2%) were male. The age of the participants ranged from 6 years to 90 years with a mean (standard deviation, SD) of 36.2 (18.0) years. Most of the participants were from the age group 20–39 years (36.0%) followed by 40–59 years (29.0%). The age of participants might not have been representative of the general population as participants were those present in the house at the time of study visit. The Tharu ethnic group was the most prevalent representing alone 24.7% of the whole population. Following the Ministry of Health classification, almost half of the population analysed belonged to the Janajati ethnic group (48%) followed by Brahmin/Chhetri (30.3%). One-third of participants were from Kailali district (30.2%) followed by Nawalparasi (13.6%) and Bardiya (10.3%). A total of 7.7% of the study participants mentioned that they had previous history of malaria.

**Table 1:** Demographic characteristics of participants, n (%)

Characteristics	n = 4057
Demographic	
Gender	
M	2564 (63.2)
F	1493 (36.8)
Age group (years)	
6–19	880 (21.7)
20–39	1461 (36.0)
40–59	1176 (29.0)
> 60	540 (13.3)
Ethnicity	
Brahman and Chhetri	
Brahman	560 (13.8)
Chhetri	719 (17.7)
Dalit	546 (13.5)
Janajati	
Janajati (other)	566 (14.0)
Chaudhary	274 (6.8)
Mahatto	102 (2.5)
Tharu	1004 (24.7)
Madhesi	98 (2.4)
Muslim	63 (1.6)
Others	125 (3.1)
District	
Banke	264 (6.5)
Bardiya	417 (10.3)
Chitwan	272 (6.7)
Dadeldhura	133 (3.3)
Kailali	1223 (30.1)
Kanchanpur	399 (9.8)
Kapilvastu	136 (3.4)
Makwanpur	137 (3.4)
Nawalparasi	553 (13.6)
Rautahat	141 (3.5)
Sindhuli	138 (3.4)
Surkhet	244 (6.0)

### G6PD phenotypes

The G6PD phenotypic status among the participants is presented in Tables 2, 3 and 4. The overall prevalence of G6PD deficiency in the study population was 3.5%, with a higher prevalence among males (4.4%) compared to females (2.1%, P < 0.01) as expected by an X-linked condition. This estimate includes 16 tests (0.4%) that were interpreted as suspected or borderline deficient. The prevalence of G6PD deficiency when classified by ethnic groups (Table 3) was highest among Janajati MOH ethnic group (overall 6.2%); in particular, in the Mahatto group (17.6%) it was significantly higher compared to any other group and it was similar in the Chaudhary (7.7%) and Tharu (7.5%), lower than the Mahatto but significantly higher compared to all the remaining groups.

**Table 2:** Distribution of G6PD phenotypes by gender

G6PD status	Male, N (%)	Female, N (%)	Total, N (%)
Deficient	112 (4.4%)	31 (2.1%)	143 (3.5%)
Normal	2442 (95.2%)	1461 (97.9%)	3903 (96.2%)
No results	10 (0.4%)	1 (0.1%)	11 (0.3%)
Total	2564 (100.0%)	1493 (100.0%)	4057 (100.0%)

**Table 3:** G6PD phenotypes by ethnic group

Ethnic groups (MOH)	Ethnic groups	Deficient, N (%)	Normal, N (%)	Total, N (%)	P
Brahman and Chhetri	Brahman	9 (1.6%)	550 (98.4%)	559	< 0.05 vs Cha, M and T
	Chhetri	7 (1.0%)	708 (99.0%)	715	< 0.05 vs Cha, M and T
Dalit	Dalit	5 (0.9%)	539 (99.1%)	544	< 0.05 vs Cha, M and T
Janajati	Janajati (other)	6 (1.1%)	560 (98.9%)	566	< 0.05 vs Cha, M and T
	Chaudhary	21 (7.7%)	253 (92.3%)	274	< 0.05 vs B, Chh, D, J and M
	Mahatto	18 (17.6%)	84 (82.4%)	102	< 0.05 vs all groups
	Tharu	75 (7.5%)	925 (92.5%)	1000	< 0.05 vs B, Chh, D, J and M
Madhesi	Madhesi	1 (1.0%)	97 (99.8%)	98	na
Muslim	Muslim	0 (0.0%)	63 (100.0%)	63	na
Others	Others	1 (0.8%)	124 (99.2%)	125	na
Total	Total	143 (3.5%)	3903 (96.5%)	4046	

*Cha* Chaudhary, *M* Mahatto, *T* Tharu, *B* Brahman, *Chh* Chhetri, *D* Dalit, *J* Janajati (other)

**Table 4:** Prevalence of G6PD deficiency by district

District	Deficient, N (%)	Normal, N (%)	Total
Banke	20 (7.6%)	243 (92.4%)	263
Chitwan	18 (6.6%)	254 (93.4%)	272
Nawalparasi	24 (4.3%)	529 (95.7%)	553
Bardiya	18 (4.3%)	399 (95.7%)	417
Kailali	49 (4.0%)	1166 (96.0%)	1215
Kapilvastu	5 (3.7%)	131 (96.3%)	136
Rautahat	4 (2.8%)	137 (97.2%)	141
Sindhuli	2 (1.4%)	136 (98.6%)	138
Kanchanpur	2 (0.5%)	395 (99.0%)	397
Surkhet	1 (0.4%)	243 (99.6%)	244
Dadeldhura	0 (0.0%)	133 (100.0%)	133
Makwanpur	0 (0.0%)	137 (100.0%)	137
Total	143 (3.5%)	3903 (96.5%)	4046

**Fig. 1:**
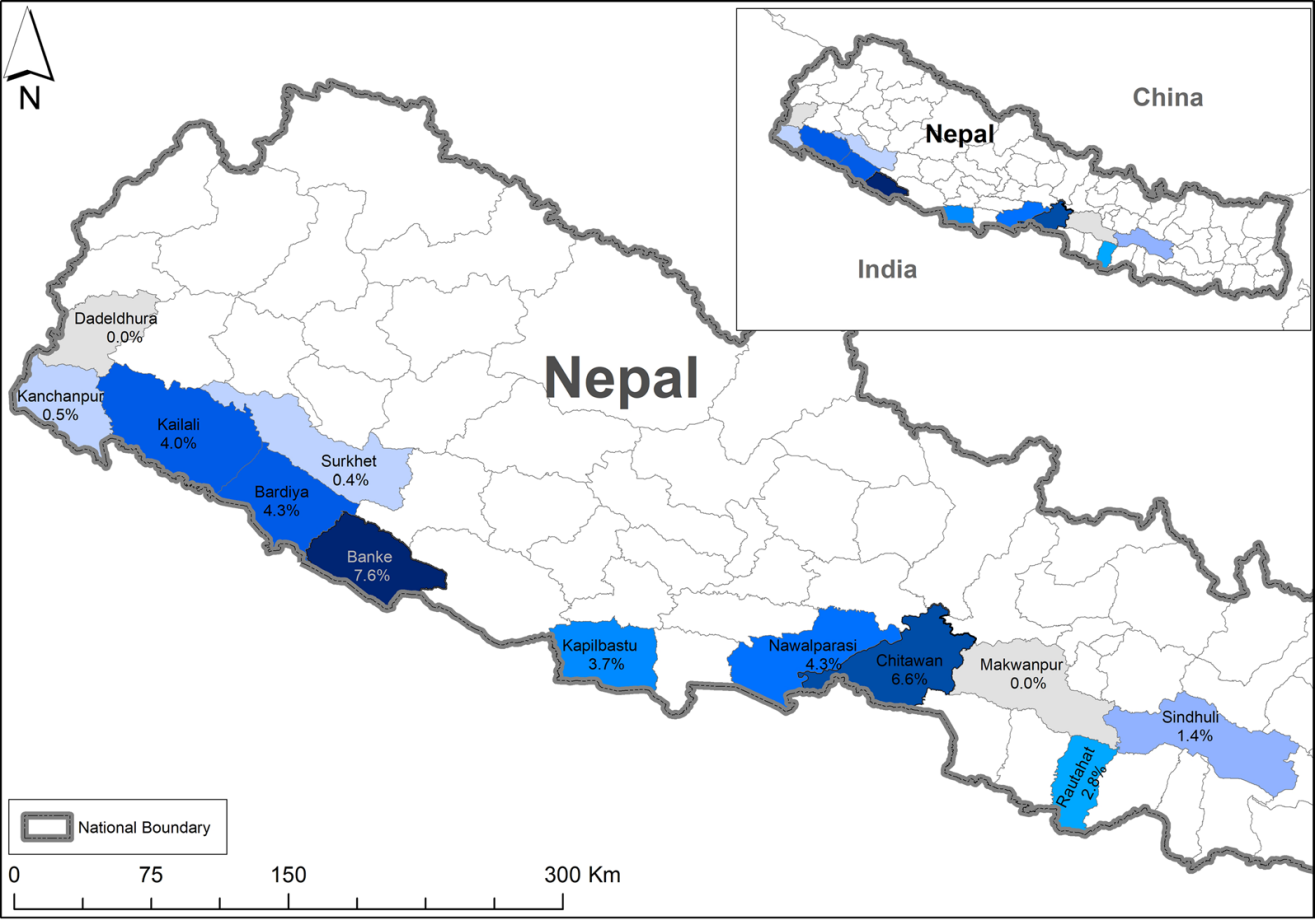
Prevalence of G6PD deficiency in malaria-endemic districts of Nepal

Prevalence of G6PD deficiency was highest in Banke district (7.6%) followed by Chitwan (6.6%), Nawalparasi and Bardiya (4.3%), and Kailali (4.0%) (Table 4 and Fig. 1). The other districts showed prevalence lower than 4% and nil in Makwanpur and Dadeldhura. When analysed by larger developmental region (Additional file 1: Table S3), G6PD deficiency prevalence ranged from 2.9% to 4.2% and was not different among the regions.

Prevalence of G6PD deficiency across districts within the same ethnic group was highly variable; the most numerous ethnic group Tharu had a prevalence ranging from 1.6% in Kanchanpur to 23.4% in Banke district. Chhetri and Brahman had a prevalence ranging from nil to 7.1% and 9.1%, respectively, in different districts (Additional file 1: Table S4 and Fig. S1).

### G6PD genotypes

Out of 143 deficient samples screened in the field, 118 had an available sample to perform genotyping. Overall the DNA quality of the samples analysed was acceptable for both standard PCR–RFLP protocol and sequencing of short-length fragments. Long-length fragments such as exon 9 and 13 required instead more work and numerous attempts of amplification and sequencing. A total of three samples could not be amplified in these two exons and one sample could not be amplified at any exon.

Among the 117 analysed samples the genotypes were distributed as shown in Table 5. The 19 samples that did not have Coimbra, Mahidol or Mediterranean mutations in exon 6 were analysed in the remaining exons. No mutations were found in the other exons and samples were therefore considered wild type. Coimbra accounted for the vast majority of mutations (74.8% of all mutated alleles), Mahidol and Mediterranean were found at same frequency (12.6% each).

**Table 5:** G6PD genotypes

	Female	Male
Coimbra		
Hemizygote	–	60
Homozygote	5	–
Heterozygote	9 (+ 3)^a^	–
Mahidol		
Hemizygote	–	10
Homozygote	–	–
Heterozygote	1 (+ 2)*	–
Mediterranean		
Hemizygote	–	11
Homozygote	–	–
Heterozygote	1 (+ 1)^a^	–
Wild type	7	10

^a^Two women with double-heterozygous genotype Coimbra/Mahidol and one with Coimbra/Mediterranean

As already seen in the prevalence of phenotypic deficiency, the cumulative allelic frequency of the three mutations was higher in the Mahatto, Chaudhary and Tharu groups who all also harboured the three mutations (Table 6). No specific geographic or ethnic distribution pattern was observed for the three mutations, with Coimbra being present in all districts where G6PD deficiency was observed (Additional file 1: Table S5) and at the higher allelic frequency in all ethnic groups with the exception of the Brahman where it was absent.

**Table 6:** Estimated allelic frequencies (%) by ethnic group

Ethnic group (MOH)	Ethnic groups	Coimbra	Mahidol	Mediterranean	Total
Brahman and Chhetri	Brahman	0.0	0.1	0.4	0.5
	Chhetri	0.2	0.0	0.0	0.2
Dalit	Dalit	0.1	0.0	0.0	0.1
Janajati	Chaudhary	4.0	0.3	0.8	5.0
	Janajati (other)	0.4	0.0	0.1	0.5
	Mahatto	8.5	1.4	2.1	12.1
	Tharu	3.9	0.6	0.2	4.7
Madhesi	Madhesi	0.7	0.0	0.0	0.7
Muslim	Muslim	0.0	0.0	0.0	0.0
Others	Others	0.0	0.6	0.0	0.6
Total	Total	1.6	0.2	0.2	2.0

### Performance of CareStart™ rapid diagnostic test

The performances of the CareStart™ rapid diagnostic test (RDT) are shown in Table 7. Although it was not possible to calculate sensitivity and specificity of the G6PD CareStart™ because the genotyping was carried out only on subjects with deficient phenotype, a relatively large number of RDT-deficient samples (12/101 and 5/16 suspected deficient) had a wild type genotype confirmed by full gene sequence. This could be due to failure in developing a strong colour in the reading window or problems in the quality of the tests. The CareStart™ G6PD test in its current formulation does not have a control band.

**Table 7:** Carestart™ RDT results by G6PD genotype

Genotype	RDT-deficient, N (%)	RDT-suspected deficient, N (%)	Total
Females			
Coimbra/Coimbra	5 (100.0%)	0	5
Coimbra/Mahidol	2 (100.0%)	0	2
Coimbra/Med	1 (100.0%)	0	1
Coimbra/WT	8 (88.9%)	1 (11.1%)	9
Mahidol/WT	0	1 (100.0%)	1
Med/WT	1 (100.0%)	0	1
Wild type	6 (85.7%)	1 (14.3%)	7
Males			
Coimbra/Y	53 (88.3%)	7 (11.7%)	60
Mahidol/Y	9 (90.0%)	1 (10.0%)	10
Med/Y	10 (90.9%)	1 (9.1%)	11
Wild type	6 (60.0%)	4 (40.0%)	10

Coimbra/WT, Mahidol/WT and Med/WT are heterozygous

## Discussion and conclusion

In the current study, the overall prevalence of G6PD deficiency was 3.5% at the population level, with a wide geographical variation (from nil to 7.6%) and across ethnicities (from nil to 17.6%). This prevalence is consistent with the worldwide prevalence (4.9%), and the prevalence in Asia (4.7%) and across much of the Indian Sub-continent (2–5%) [5, 12]. In a similar survey in Nepal in 2013, the prevalence of G6PD deficiency was higher, especially in the most eastern districts of the Terai regions [25] among Rajbanshi (11.7%, classified as Janajati by MOH) and Tharus (5.6%); prevalence was only 1.4% among Brahmins and Chhetris. A similar prevalence among Brahmins and Chhetris was found in the current study and higher prevalence in Tharus, Mahatto and Chaudhary of the Janajati group.

The sensitivity and specificity of the G6PD CareStart™ RDT could not be formally assessed in this study; the inconsistencies observed in this context hint to a more general consideration on the necessity to use fully validated and quality-controlled G6PD tests for malaria elimination strategies to be effective.

The three G6PD variants found had a broad distribution geographically and among ethnicities in the studied districts. Interestingly, Mahidol is a mutation found mainly in the most western countries of Southeast Asia (Myanmar and Thailand [32, 33]) and Coimbra is a mutation found at low frequencies predominantly in the Indian region of Uttar Pradesh [34], in Tharus living in India [35] and rarely in Myanmar [36] and Malaysia [37]. The relatively high allelic frequency of Coimbra variant found in the Mahatto (8.5%) and in the Tharu (3.9%) in the current study seems to exceed all previous published reports. The Mediterranean variant is found in Europe and Asia, being the most frequent G6PD mutation in India among the caste groups [38, 39]. Overall the genetic data of the most numerous ethnic group in the current study (the Tharus) align to previous evidence of their dual Southeast Asian and Indian ancestry [40, 41].

Primaquine and tafenoquine are the only currently available drugs effective against hypnozoites of *P. vivax* [12]. The adverse effects of 8-aminoquinolines among G6PD-deficient individuals are widely documented [2, 11, 42] although more evidence is still needed on haemolytic risk associated with different regimens and populations [12, 43]. The WHO has recommended G6PD screening among populations in regions where G6PD deficiency prevalence is relatively high, and *P. vivax* radical cure treatment regimens be based on the level of G6PD activity [44, 45].

The findings of the current study have therefore very clear programmatic and policy-making implications. They show that G6PD deficiency is present broadly in the Terai regions of Nepal and is caused by mutations associated to described primaquine-induced haemolysis. Some ethnic groups are more affected but the prevalence of deficiency within the same ethnicity varies widely in different districts. Since the risk of haemolysis due to G6PD deficiency cannot be excluded on the basis of the geographic region or ethnicity alone, G6PD testing will be always necessary before deployment of primaquine. The EDCD in Nepal has provided directives for G6PD screening among the population at health facility level [46]. However, its implementation in the routine health system is still not in practice. Further evidence might also be required in assessing the cost-effectiveness of introducing screening kits. In the context of the available evidence and following the WHO recommendation, stakeholders, including government and academia are invited to: (i) conduct further studies in malaria-endemic as well as non-endemic districts to determine the prevalence, genetic variant of G6PD and the risk population; (ii) ensure the availability of G6PD screening kits; (iii) demand training of health workers to ensure that the revised malaria treatment protocol is followed across primary health facilities; (iv) develop a pharmacovigilance system to report the adverse events with the use of primaquine; and, (v) manage the adverse events associated with the drug through proper clinical training of health workers and availability of referral system.

## Supplementary information

**Additional file 1.** Tables S1–S5 and Figure S1.

## Data Availability

The datasets used and/or analysed during the current study are available from the corresponding author on reasonable request.
