# Supplementary material for: G6PD deficiency in malaria endemic areas of Nepal
Source: Malar J. 2020 Aug 12;19:287. doi: 10.1186/s12936-020-03359-6 (PMC7425560; doi:10.1186/s12936-020-03359-6)

**Additional material**

**Table S1. Primer sequences**

| Exon 2 | PCR_G6PD_Ex2_F  PCR_G6PD_Ex2_R | 5’-TGAAGGCTGCCTAGGAGAGA-3’  5’-CAGGTAGAGCCGGGATGAT-3’ |
| --- | --- | --- |
| Exon 3-4 | PCR_G6PD_Ex3-4_F  PCR_G6PD_Ex3-4_R | 5’-TGTCCCCAGCCACTTCTAA-3’  5’-GGAGAGGAGGAGAGCATCC-3’ |
| Exon 5 | PCR_G6PD_Ex5_F  PCR_G6PD_Ex5_R | 5’- CGGGGACACTGACTTCTG -3’  5’-ACGCTGCCACCTTGTGGT-3’ |
| Exon 6-7 | PCR_G6PD_Ex6-7_F  PCR_G6PD_Ex6-7_R | 5’-ACACAAGGCACGGGAGGT-3’  5’-GAGGAGCTCCCCCAAGATAG-3’ |
| Exon 8 | PCR_G6PD_Ex8_F  PCR_G6PD_Ex8_R | 5’-CCCTTGAACCAGGTGAACAG-3’  5’-TCAGTGCCTCGTCACAGATG-3’ |
| Exon 9 | PCR_G6PD_Ex9_F  PCR_G6PD_Ex9_R | 5’-CCTGAGGGCTGCACATCT-3’  5’-GTGCGTGAGTGTCTCAGTGG-3’ |
| Exon 10 | PCR_G6PD_Ex10-12_F  G6PD_EX10_R | 5’-TGAGACACTCACGCACTGGT-3’  5’-CTGCCACCATGTGGA GTC-3’ |
| Exon 11-12 | G6PD-EX11_F  PCR_G6PD_Ex10-12_R | 5’-GACTCCACATGGTGGCAG-3’  5’-TGAGGTAGCTCCACCCTCAC-3’ |
| Exon 13 | PCR_G6PD_Ex13_F  PCR_G6PD_Ex13_R | 5’-TTATGGCAGGTGAGGAAAGG-3’  5’-GAAGTGGGTCCTCAGGGAAG-3’ |

**Table S2. Annealing Temperatures**

| **Amplicon** | **Annealing temperature** |
| --- | --- |
| Exon 2 | 63°C |
| Exon 3-4 | 63°C |
| Exon 5 | 61°C |
| Exon 6-7 | 61°C |
| Exon 8 | 64°C |
| Exon 9 | 64°C |
| Exon 10 | Touch-down 70°C to 63°C; 63°C |
| Exon 11-12 | 64°C |
| Exon 13 | Touch-down 70°C to 63°C; 63°C |

**Table S3. G6PD phenotypes by larger development regions**

| Larger region | Deficient | Normal | No Result | Total |
| --- | --- | --- | --- | --- |
| Far-West | 51 (2.9%) | 1694 (96.5%) | 10 (0.6%) | 1755 |
| Mid-West | 39 (4.2%) | 885 (95.7%) | 1 (0.1%) | 925 |
| West | 29 (4.2%) | 660 (95.8%) | 0 | 689 |
| Central | 24 (3.5%) | 664 (96.5%) | 0 | 688 |
| Total | 143 (3.5%) | 3903 (96.2%) | 11 (0.3%) | 4057 |

**Table S4. G6PD phenotypes by Ethnic group and district**

| Ethnic group | District | G6PD deficient | G6PD normal | Total |
| --- | --- | --- | --- | --- |
| Brahman | Banke | 2 | 20 | 22 |
|  |  | 9.1% | 90.9% | 100.0% |
|  | Bardiya | 0 | 20 | 20 |
|  |  | 0.0% | 100.0% | 100.0% |
|  | Chitwan | 0 | 72 | 72 |
|  |  | 0.0% | 100.0% | 100.0% |
|  | Dadeldhura | 0 | 26 | 26 |
|  |  | 0.0% | 100.0% | 100.0% |
|  | Kailali | 1 | 120 | 121 |
|  |  | 0.8% | 99.2% | 100.0% |
|  | Kanchanpur | 0 | 63 | 63 |
|  |  | 0.0% | 100.0% | 100.0% |
|  | Kapilvastu | 3 | 78 | 81 |
|  |  | 3.7% | 96.3% | 100.0% |
|  | Makwanpur | 0 | 8 | 8 |
|  |  | 0.0% | 100.0% | 100.0% |
|  | Nawalparasi | 3 | 121 | 124 |
|  |  | 2.4% | 97.6% | 100.0% |
|  | Rautahat | 0 | 8 | 8 |
|  |  | 0.0% | 100.0% | 100.0% |
|  | Sindhuli | 0 | 2 | 2 |
|  |  | 0.0% | 100.0% | 100.0% |
|  | Surkhet | 0 | 12 | 12 |
|  |  | 0.0% | 100.0% | 100.0% |
| Chaudhary | Banke | 0 | 5 | 5 |
|  |  | 0.0% | 100.0% | 100.0% |
|  | Bardiya | 0 | 4 | 4 |
|  |  | 0.0% | 100.0% | 100.0% |
|  | Chitwan | 0 | 9 | 9 |
|  |  | 0.0% | 100.0% | 100.0% |
|  | Kailali | 18 | 165 | 183 |
|  |  | 9.8% | 90.2% | 100.0% |
|  | Kanchanpur | 0 | 7 | 7 |
|  |  | 0.0% | 100.0% | 100.0% |
|  | Nawalparasi | 2 | 4 | 6 |
|  |  | 33.3% | 66.7% | 100.0% |
|  | Rautahat | 1 | 59 | 60 |
|  |  | 1.7% | 98.3% | 100.0% |
| Chhetri | Banke | 2 | 26 | 28 |
|  |  | 7.1% | 92.9% | 100.0% |
|  | Bardiya | 0 | 28 | 28 |
|  |  | 0.0% | 100.0% | 100.0% |
|  | Chitwan | 1 | 14 | 15 |
|  |  | 6.7% | 93.3% | 100.0% |
|  | Dadeldhura | 0 | 80 | 80 |
|  |  | 0.0% | 100.0% | 100.0% |
|  | Kailali | 1 | 274 | 275 |
|  |  | 0.4% | 99.6% | 100.0% |
|  | Kanchanpur | 0 | 111 | 111 |
|  |  | 0.0% | 100.0% | 100.0% |
|  | Kapilvastu | 0 | 9 | 9 |
|  |  | 0.0% | 100.0% | 100.0% |
|  | Makwanpur | 0 | 6 | 6 |
|  |  | 0.0% | 100.0% | 100.0% |
|  | Nawalparasi | 3 | 42 | 45 |
|  |  | 6.7% | 93.3% | 100.0% |
|  | Rautahat | 0 | 2 | 2 |
|  |  | 0.0% | 100.0% | 100.0% |
|  | Sindhuli | 0 | 37 | 37 |
|  |  | 0.0% | 100.0% | 100.0% |
|  | Surkhet | 0 | 79 | 79 |
|  |  | 0.0% | 100.0% | 100.0% |
| Dalit | Banke | 0 | 14 | 14 |
|  |  | 0.0% | 100.0% | 100.0% |
|  | Bardiya | 1 | 22 | 23 |
|  |  | 4.3% | 95.7% | 100.0% |
|  | Chitwan | 0 | 3 | 3 |
|  |  | 0.0% | 100.0% | 100.0% |
|  | Dadeldhura | 0 | 16 | 16 |
|  |  | 0.0% | 100.0% | 100.0% |
|  | Kailali | 2 | 202 | 204 |
|  |  | 1.0% | 99.0% | 100.0% |
|  | Kanchanpur | 0 | 92 | 92 |
|  |  | 0.0% | 100.0% | 100.0% |
|  | Kapilvastu | 0 | 2 | 2 |
|  |  | 0.0% | 100.0% | 100.0% |
|  | Makwanpur | 0 | 10 | 10 |
|  |  | 0.0% | 100.0% | 100.0% |
|  | Nawalparasi | 1 | 48 | 49 |
|  |  | 2.0% | 98.0% | 100.0% |
|  | Rautahat | 0 | 19 | 19 |
|  |  | 0.0% | 100.0% | 100.0% |
|  | Sindhuli | 0 | 5 | 5 |
|  |  | 0.0% | 100.0% | 100.0% |
|  | Surkhet | 1 | 106 | 107 |
|  |  | 0.9% | 99.1% | 100.0% |
| Janajati | Banke | 0 | 6 | 6 |
|  |  | 0.0% | 100.0% | 100.0% |
|  | Bardiya | 0 | 14 | 14 |
|  |  | 0.0% | 100.0% | 100.0% |
|  | Chitwan | 0 | 66 | 66 |
|  |  | 0.0% | 100.0% | 100.0% |
|  | Dadeldhura | 0 | 4 | 4 |
|  |  | 0.0% | 100.0% | 100.0% |
|  | Kailali | 1 | 53 | 54 |
|  |  | 1.9% | 98.1% | 100.0% |
|  | Kanchanpur | 1 | 45 | 46 |
|  |  | 2.2% | 97.8% | 100.0% |
|  | Kapilvastu | 0 | 9 | 9 |
|  |  | 0.0% | 100.0% | 100.0% |
|  | Makwanpur | 0 | 104 | 104 |
|  |  | 0.0% | 100.0% | 100.0% |
|  | Nawalparasi | 2 | 136 | 138 |
|  |  | 1.4% | 98.6% | 100.0% |
|  | Rautahat | 0 | 2 | 2 |
|  |  | 0.0% | 100.0% | 100.0% |
|  | Sindhuli | 2 | 87 | 89 |
|  |  | 2.2% | 97.8% | 100.0% |
|  | Surkhet | 0 | 34 | 34 |
|  |  | 0.0% | 100.0% | 100.0% |
| Madhesi | Banke | 1 | 64 | 65 |
|  |  | 1.5% | 98.5% | 100.0% |
|  | Chitwan | 0 | 9 | 9 |
|  |  | 0.0% | 100.0% | 100.0% |
|  | Kailali | 0 | 3 | 3 |
|  |  | 0.0% | 100.0% | 100.0% |
|  | Nawalparasi | 0 | 10 | 10 |
|  |  | 0.0% | 100.0% | 100.0% |
|  | Rautahat | 0 | 11 | 11 |
|  |  | 0.0% | 100.0% | 100.0% |
| Mahatto | Chitwan | 17 | 62 | 79 |
|  |  | 21.5% | 78.5% | 100.0% |
|  | Nawalparasi | 1 | 22 | 23 |
|  |  | 4.3% | 95.7% | 100.0% |
| Muslim | Banke |  | 49 | 49 |
|  |  |  | 100.0% | 100.0% |
|  | Chitwan |  | 14 | 14 |
|  |  |  | 100.0% | 100.0% |
| Tharu | Banke | 15 | 49 | 64 |
|  |  | 23.4% | 76.6% | 100.0% |
|  | Bardiya | 17 | 301 | 318 |
|  |  | 5.3% | 94.7% | 100.0% |
|  | Kailali | 26 | 326 | 352 |
|  |  | 7.4% | 92.6% | 100.0% |
|  | Kanchanpur | 1 | 61 | 62 |
|  |  | 1.6% | 98.4% | 100.0% |
|  | Kapilvastu | 2 | 28 | 30 |
|  |  | 6.7% | 93.3% | 100.0% |
|  | Makwanpur | 0 | 1 | 1 |
|  |  | 0.0% | 100.0% | 100.0% |
|  | Nawalparasi | 11 | 128 | 139 |
|  |  | 7.9% | 92.1% | 100.0% |
|  | Rautahat | 3 | 31 | 34 |
|  |  | 8.8% | 91.2% | 100.0% |
| Others | Banke | 0 | 10 | 10 |
|  |  | 0.0% | 100.0% | 100.0% |
|  | Bardiya | 0 | 10 | 10 |
|  |  | 0.0% | 100.0% | 100.0% |
|  | Chitwan | 0 | 5 | 5 |
|  |  | 0.0% | 100.0% | 100.0% |
|  | Dadeldhura | 0 | 7 | 7 |
|  |  | 0.0% | 100.0% | 100.0% |
|  | Kailali | 0 | 23 | 23 |
|  |  | 0.0% | 100.0% | 100.0% |
|  | Kanchanpur | 0 | 16 | 16 |
|  |  | 0.0% | 100.0% | 100.0% |
|  | Kapilvastu | 0 | 5 | 5 |
|  |  | 0.0% | 100.0% | 100.0% |
|  | Makwanpur | 0 | 8 | 8 |
|  |  | 0.0% | 100.0% | 100.0% |
|  | Nawalparasi | 1 | 18 | 19 |
|  |  | 5.3% | 94.7% | 100.0% |
|  | Rautahat | 0 | 5 | 5 |
|  |  | 0.0% | 100.0% | 100.0% |
|  | Sindhuli | 0 | 5 | 5 |
|  |  | 0.0% | 100.0% | 100.0% |
|  | Surkhet | 0 | 12 | 12 |
|  |  | 0.0% | 100.0% | 100.0% |

**Table S5. Estimated allelic frequencies (%) by district**

|  | Coimbra | Mahidol | Mediterranean | total |
| --- | --- | --- | --- | --- |
| Banke | 4.5 | 0.6 | 0.0 | 5.1 |
| Chitwan | 3.0 | 0.5 | 0.8 | 4.4 |
| Bardiya | 2.3 | 0.2 | 0.2 | 2.7 |
| Nawalparasi | 1.7 | 0.7 | 0.1 | 2.5 |
| Kailali | 1.6 | 0.2 | 0.4 | 2.2 |
| Rautahat | 2.1 | 0.0 | 0.0 | 2.1 |
| Sindhuli | 1.1 | 0.0 | 0.0 | 1.1 |
| Kapilvastu | 0.6 | 0.0 | 0.6 | 1.1 |
| Dadeldhura, Kanchanpur, Makwanpur, Surkhet | 0.0 | 0.0 | 0.0 | 0.0 |
| Total | 1.6 | 0.2 | 0.2 | 2.0 |

**Figure S1. G6PD phenotypes by district and ethnic group in the four most numerous ethnicities**


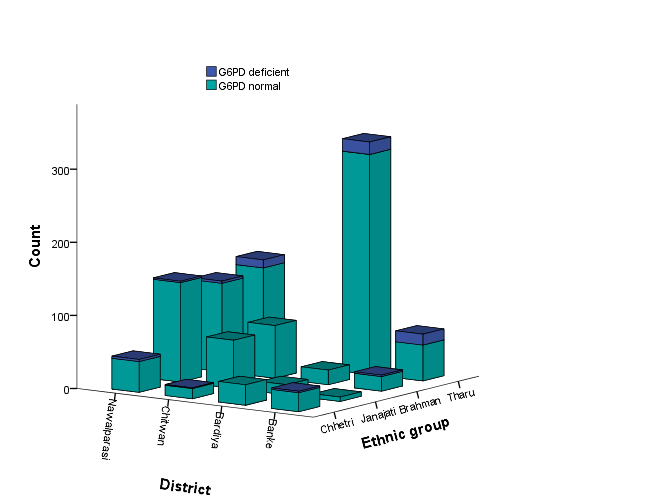

Supplement: Supplementary file 1 — Additional file 1. Tables S1–S5 and Figure S1. [file 12936_2020_3359_MOESM1_ESM.docx]
